# Novel type of references for weight aligned for onset of puberty – using the QEPS growth model

**DOI:** 10.1186/s12887-021-02954-z

**Published:** 2021-11-13

**Authors:** Kerstin Albertsson-Wikland, Aimon Niklasson, Lars Gelander, Anton Holmgren, Andreas F. M. Nierop

**Affiliations:** 1 Department of Physiology/Endocrinology, Institute of Neuroscience and Physiology, Sahlgrenska Academy, University of Gothenburg, 405 30 Gothenburg, SE Sweden; 2 Göteborg Pediatric Growth Research Center, Department of Pediatrics, Institute of Clinical Sciences, Sahlgrenska Academy, University of Gothenburg, Gothenburg, Sweden; 3 Department of Pediatrics, Halmstad Hospital, Halmstad, Sweden; 4 Muvara bv, Multivariate Analysis of Research Data, Leiderdorp, The Netherlands

**Keywords:** Pubertal growth, Personalized growth, Biological age, SDS, Weight status, Longitudinal BMI-selection; reference population

## Abstract

**Background:**

Growth references are traditionally constructed relative to chronological age, despite inter-individual variations in pubertal timing. A new type of height reference was recently developed allowing growth to be aligned based on onset of pubertal height growth. We here aim to develop a corresponding reference for pubertal weight.

**Methods:**

To model QEPS-weight, 3595 subjects (1779 girls) from GrowUp_1974_Gothenburg and GrowUp_1990_Gothenburg were used. The QEPS-height-model was transformed to a corresponding QEPS-weight-model; thereafter, QEPS-weight was modified by an individual, constitutional weight-height-factor. Longitudinal weight and length/height measurements from 1418 individuals (698 girls) from GrowUp_1990_Gothenburg were then used to create weight references aligned for height at pubertal onset (the age at 5% of P-function growth, AgeP5). GrowUp_1974_Gothenburg subgroups based on pubertal timing, stature at pubertal onset, and childhood body composition were assessed using the references.

**Results:**

References (median, SDS) for total weight (QEPS-functions), weight specific to puberty (P-function), and weight gain in the absence of specific pubertal growth (basic weight, QES-functions), allowing alignment of individual growth based on age at pubertal onset. For both sexes, basic weight was greater than average for late maturing, tall and high-BMI subgroups. The P-function-related weight was greater than average in short and lower than average in tall children, in those with high BMI, and in girls but not boys with low BMI.

**Conclusions:**

New pubertal weight references allow individual variations in pubertal timing to be taken into consideration when evaluating growth. When used together with the comparable pubertal height reference, this will improve growth monitoring in clinical practice for identifying abnormal growth and serve as a valuable research tool providing insight into human growth.

**Supplementary Information:**

The online version contains supplementary material available at 10.1186/s12887-021-02954-z.

## Background

The growth curve of an individual child reflects both the physical and psychosocial wellbeing of that child. According to the World Health Organization (WHO), every child has the right to grow and be healthy, and growth references or standards are crucial tools for monitoring such growth [1]. It is therefore important that repeated height and weight measurements are obtained for every child within a healthcare setting, and plotted alongside established growth references, preferably using a computerized system [1–3]. In order to be optimal, references should be constructed using data from children growing optimally. Height references should be updated regularly in countries where the population is still experiencing a secular trend for height to increase over time, as was recently done in Sweden [4]. Similarly, in some countries, updates to weight references should consider that weight development may be unhealthy in children and adolescents owing to the ongoing obesity epidemic. This issue was addressed in the newly updated references for weight in Sweden by omitting individuals with extreme obesity from the longitudinal BMI data set used to develop the tool [5].

Growth references, both for height and weight, are traditionally presented in relation to chronological age. This remains the case despite the understanding that there is a broad variation in biological age between individuals, especially during puberty [3, 6]. As a result of the reliance on chronological age, these references are of limited use for detecting abnormal growth during adolescence. Since the year 2000, Swedish healthcare systems have used references that incorporate an estimate of ongoing, basic, childhood growth based on the childhood growth function from the ICP-growth model [7–10]. Use of this growth function has proved helpful in distinguishing ‘healthy’ late maturers from individuals with growth failure owing to an underlying condition or disease. When developing the most recent Swedish height references using a cohort of children born in 1990, this type of ‘prepubertal’ height reference showing ongoing basic growth was produced using the QE-functions of the QEPS-model [11, 12]. The QEPS-model describes individual growth using four mathematical functions: a Q (Quadratic) and E (Exponential) arising before birth and resulting in the basic growth, to which a specific pubertal growth function, P, is added; growth is ended by an S (stop) function [11]. We were recently able to develop and publish a completely new type of height reference describing growth resulting from the specific pubertal growth function of the QEPS model. This reference allows the height of the individual to be aligned relative to the onset of pubertal growth, and provides information on total growth, as well as separating growth that is a specific result of puberty from ongoing, basic growth that is continuing during the pubertal years [13, 14]. As changes in height and weight should ideally be evaluated simultaneously, there remains a need for a corresponding reference for both prepubertal and pubertal weight.

The aim of this study was to use the QEPS-model to develop new references for weight during adolescence taking biological maturation into account. The weight references will be constructed using data from a homogeneous, longitudinally followed population of healthy children born at term to non-smoking mothers and Nordic parents, selected from the GrowUp_1990_Gothenburg cohort born in Sweden in around 1990 [5]. It will be possible to use the weight references to evaluate pubertal growth in a child, irrespective of the chronological age at which pubertal growth begins in that individual. Separate references will be available for (i) total weight (modeled by the Q, E, P and S functions), (ii) weight gain that is specific to puberty (by the P function), and (iii) weight gain in the absence of growth specific to puberty (basic weight growth) (by the Q, E and S functions). We will also explore the usefulness of the new references for monitoring weight by analyzing subgroups of children from the GrowUp_1974_Gothenburg cohort categorized according to the timing of puberty (early, average, late), height at start of puberty (tall, short) and body composition during childhood (high body mass index (BMI), low BMI), as used for exploring the comparable pubertal height reference [14].

## Methods

### Materials

#### Subset selection from GrowUp_1974 &1990_Gothenburg cohorts for modelling QEPS weight

Data were retrieved from two community based observational growth studies conducted in high schools in Gothenburg, Sweden: GrowUp_1974_Gothenburg and GrowUp_1990_Gothenburg [9, 15, 16]. Individuals with extreme BMI values relative to the GrowUp_1974_Gothenburg BMI_SDS_ reference according to the criteria earlier defined for population used for weight references versus chronological age [5], were excluded from both cohorts, leaving 2177 individuals (1081 girls) from GrowUp_1974_Gothenburg and 1418 individuals (698 girls) from GrowUp_1990_Gothenburg. The latter cohort served as the reference population for the recently published LMS weight, weight-for-height, and BMI references [5]. Both cohorts were combined to form a group of 3595 subjects (1779 girls) which was used to construct the QEPS weight model needed for developing the references.

#### Reference population from GrowUp_1990_Gothenburg cohort

The QEPS weight reference population included 1418 individuals (698 girls) from the GrowUp_1990_Gothenburg cohort; this was the same cohort used to create the previously published LMS total weight, weight-for-height, and BMI references (see Supplemental Table S1 of [5]). Children in this cohort were healthy (see Table 1 of [5]), born between 1989 and 1991 at full term (gestational age (GA) 37–43 weeks) in Sweden to Nordic parents with non-smoking mothers, and had information available on longitudinal growth until adult height. For more information see Albertsson-Wikland et al. [5, 13].

#### Subgroups in the GrowUp_1974_Gothenburg cohort used for exploring the novel reference

Data from subgroups of healthy children from the GrowUp_1974_Gothenburg cohort (2177 subjects; 1081 girls) were used in order to explore the utility of the new pubertal weight reference as a research tool. These investigations looked in turn at the impact of grouping children according to age at onset of puberty, height at onset of puberty and childhood BMI. *According to pubertal age:* early (<− 1.5 yrs), average (±0.25 yrs) or late (> + 1.5 yrs) onset of puberty (see Supplemental Table S3 of [14]); when age at TPHV (PHV from the total height growth curve) was used to estimate pubertal age. *According to height:* tall (> + 1.5SDS) or short (<− 1.5SDS) stature at onset of puberty. A*ccording to childhood BMI:* high BMI (> + 1.5SDS) or low BMI (<− 1.5SDS).

### QEPS weight method

The QEPS height model [11], was first transformed to a corresponding QEPS weight model. The QEPS weight model is expressed in kg^0.5^, because only in kg^0.5^ it was possible to obtain an adequate fit for the additive QEPS weight model with *wT* = *wQ* + *wE* + *wP* − *wS*. In kg the additive property of the QEPS weight functions is lost. If appropriate in figures we show weight in kg at the left axis and weight in kg^0.5^ at the right axis. For detailed information see Supplemental for QEPS-weight model, including Supplemental Fig. S1, S2, S3. QEPS weight was thereafter modified by an individual constitutional factor, a so-called weight-height-factor (*WHF*): WHF = 0 stands for a ‘normal body constitution’, *WHF* > 0 for a heavier than normal and *WHF* < 0 for a leaner than normal body constitution. Traditional references for total and prepubertal weight according to chronological age were computed in two steps comparable to the ‘QEPS method used for the references’ section in Supplement of [13], but with QEPS height functions replaced by corresponding QEPS weight functions fitted on observed individual weight data, referred to as the QEPS weight method.

We compared the total weight references obtained using the QEPS weight method with the previously published weight reference obtained by applying the LMS method [5]. Both references were generated using data from the same population, Supplemental Fig. S4. The two mean reference curves were similar; however, variance was smaller for the QEPS-derived compared with the LMS-derived reference, since it was based on fitted weight functions, excluding all sorts of weight error, whereas the LMS-derived reference was fitted on the observed weight measurements, including such error.

Thereafter, a new type of reference was generated for pubertal weight designed so that growth was aligned according to onset of puberty for the individual, identified based on height-specific *P*-function growth, defined as *AgeP5*, when 5% of height *Pmax* was obtained, exactly as used for the pubertal height references (Supplemental Fig. S2 [14]) [12]. All longitudinal QEPS weight functions for individuals in the reference population were aligned according to height *AgeP5.* For more information see Supplement.

## Results

### Total and prepubertal/basic weight references vs chronological age

**Fig. 1:**
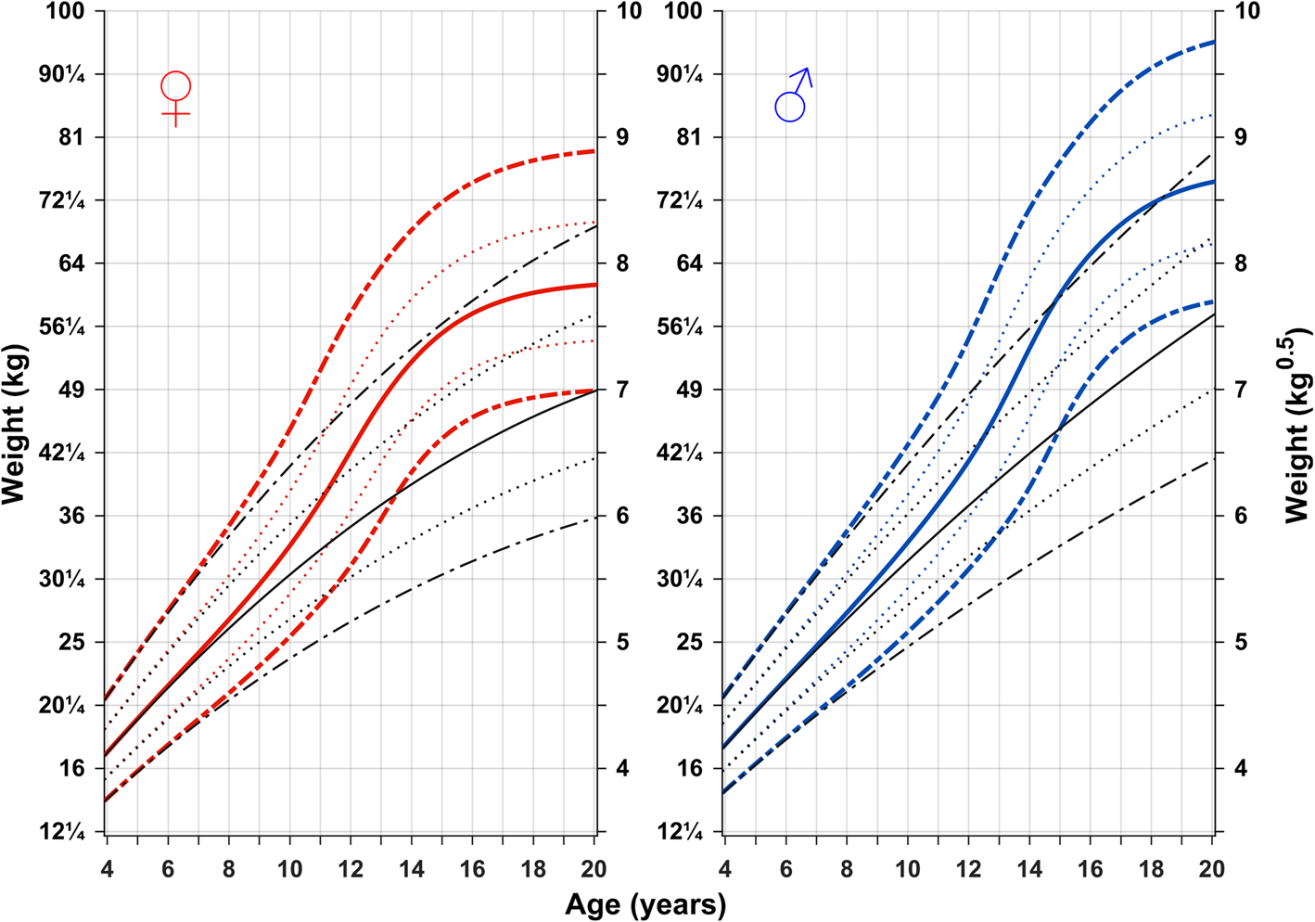
Chronological age reference for total and basic growth. References for weight (total weight and weight gained independently of puberty (basic growth = QES-function)) for girls (left) and boys (right) aged 4–20 years. Mean *total weight* (red/blue solid line, ±1SDS (red/blue dotted line), and ± 2SDS (red/blue dashed line) and mean *basic weight* (black)

Figure 1 shows the novel weight reference in kg (left axis) and in kg^0.5^ (right axis) according to chronological age for girls and boys aged 4–20 years. Total weight is shown in color, with weight gained independently of puberty (the prepubertal, basic (QES-function) weight reference) shown in black.

### Weight references aligned for onset of puberty

**Fig. 2:**
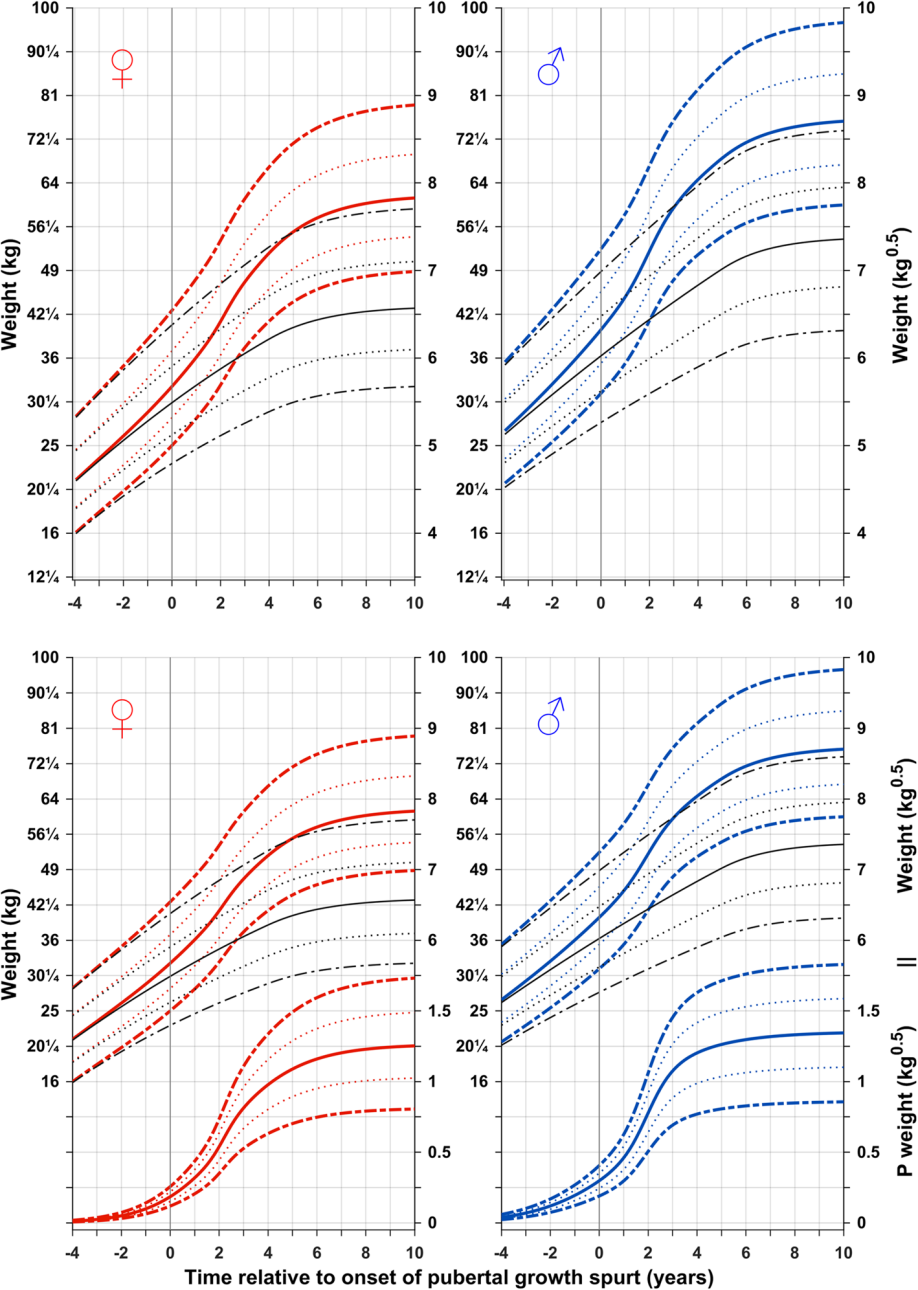
References in kg (left axis) and in kg^0.5^ (right axis) for total weight and the prepubertal/basic weight (weight gained independently of puberty (QES-function) for girls (red, left) and boys (blue, right). Curves are aligned for age at onset of pubertal growth spurt. Mean *total weight* (red/blue solid line, ±1SDS (red/blue dotted line), and ± 2SDS (red/blue dashed line) and mean *basic weight* (black). References shown in Fig. 2 upper panel with the addition of a puberty-specific weight reference showing weight gain resulting from the P-function of the QEPS (P weight). *Specific P-function-derived weight* as mean, ±1SDS (dotted line), and ± 2SDS (dashed line). The individual onset of puberty was identified and aligned based on the age at which 5% (AgeP5) of the total specific P-function growth (Pmax) had occurred

Figure 2 shows the reference for total (QEPS) and basic (QES-function) weight in kg (left axis) and kg^0.5^ (right axis) for girls and boys aligned according to the onset of puberty, estimated as AgeP5. In Fig. 2 lower panel, specific P-function growth has been included alongside the curves shown in Fig. 2 upper panel. To capture changes in weight relative to the acceleration in height that occurs during puberty, weight references in both figures are depicted from 4 years before to 10 years after the onset of the pubertal growth spurt.

### Rational for a puberty-aligned weight reference

**Fig. 3:**
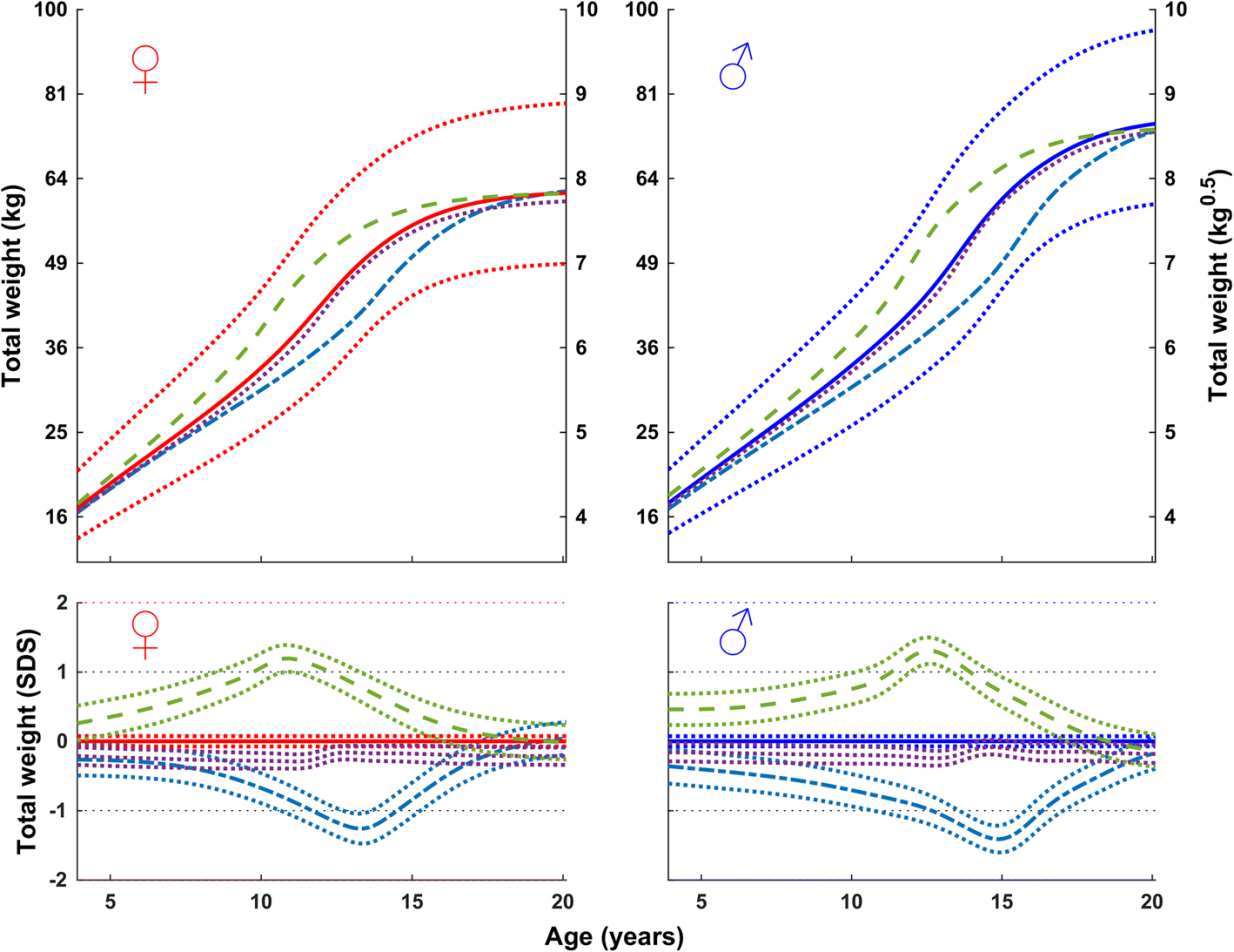
Mean total weight (in kg, top; in SDS with 95% CI, bottom) according to chronological age for girls (left) and boys (right) from the GrowUp_1974_ Gothenburg cohort grouped according to onset of puberty (early, <− 1.5 yrs. (- - -), average, ±0.25 yrs. (• • •), and late, > + 1.5 yrs. (- — -)). Data are visualized alongside the reference for total mean weight (thick solid lines) and ± 2SDS (dotted red (left) and blue (right) lines)

Figure 3 shows total weight relative to chronological age for subgroups of girls and boys with an early, average, and late onset of puberty. As expected, weight gain occurred sooner than average in children with an early onset of puberty, and later than average in children with a late onset of puberty. Thus, highlightening the inadequate usefulness for many adolescents of an ordinary chronological age weight reference due to the broad variation in chronological age at pubertal maturation within a population.

### Exploring pubertal weight in subgroups from the GrowUp_1974_Gothenburg cohort

#### Timing of puberty

**Fig. 4:**
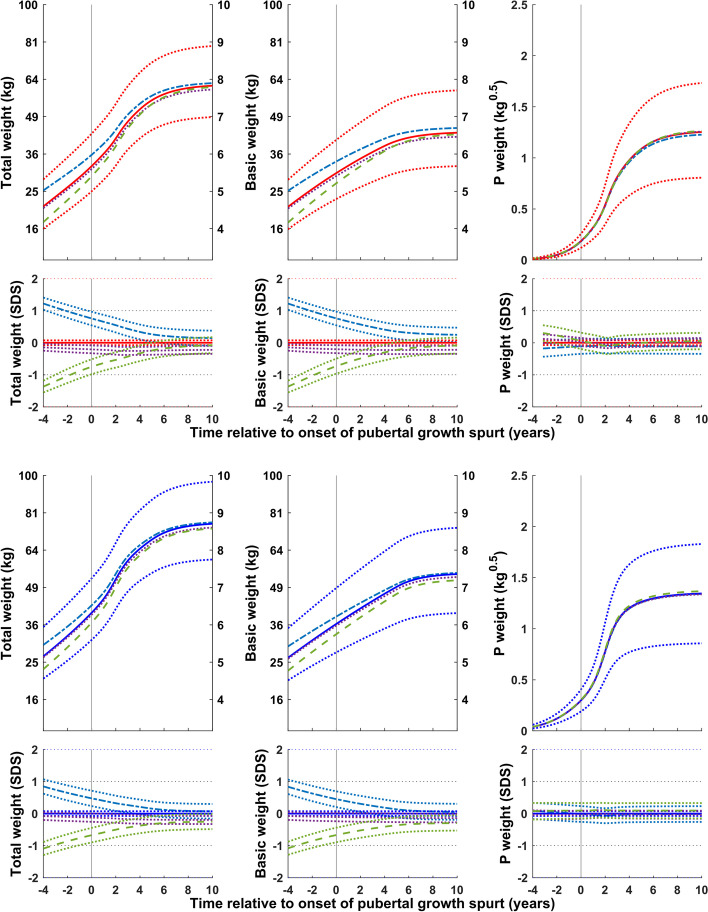
Mean total, basic and puberty-specific weight gain (in kg, top; in SDS with 95% CI, bottom) relative to onset of puberty for girls (red at the top) and boys (blue at the bottom) grouped according to onset of puberty (early, <− 1.5 yrs. (- - -), average, ±0.25 yrs. (• • •), and late, > + 1.5 yrs. (- — -)). Mean total weight (left panels), basic weight growth (middle panels), and specific pubertal weight growth (right panels) from the GrowUp_1974_Gothenburg cohort. Curves are aligned for age at onset of pubertal growth spurt. Data are visualized alongside the new reference for total mean weight (thick red or blue solid lines) and ± 2SDS (dotted blue lines)

Figure 4 shows weight (total (QEPS), basic (QES) and pubertal (P-function)) relative to the onset of puberty for subgroups of girls and boys with an early, average, and late onset of puberty. The basic component of weight gain was lower than average in the early puberty group and higher than average in the late puberty group. Specific weight gain during puberty was similar for all three groups. Total weight gain was similar in all groups.

#### Stature

**Fig. 5:**
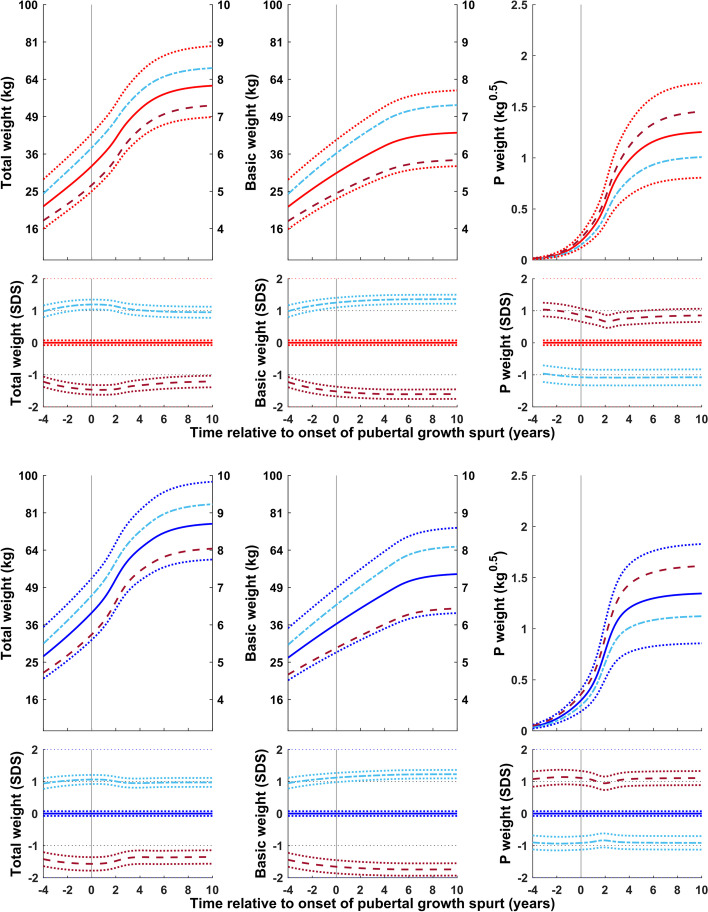
Mean total, basic and puberty-specific weight gain (in kg, top; in SDS with 95% CI, bottom) relative to the onset of puberty for girls (red at the top) and boys (blue at the bottom) according to height at the onset of puberty (tall, > + 1.5 SDS (- — -) and short, <− 1.5 SDS (- - -)). Mean total weight (left panels), basic weight growth (middle panels), and specific pubertal weight growth (right panels) from the GrowUp_1974_Gothenburg cohort. Curves are aligned for age at onset of pubertal growth spurt. Data are visualized alongside the new reference for total mean weight (thick red or blue solid lines) and ± 2SDS (dotted red or blue lines)

Figure 5 shows weight (total (QEPS), basic (QES) and pubertal (P-function)) for girls and boys according to height at the onset of puberty. The basic component of weight gain was higher than average in girls and boys classed as tall at the onset of puberty, and lower than average in girls and boys classed as short. In contrast, specific weight gain during puberty was lower than average for the tall group and higher than average for the short group.

#### Degree of BMI

**Fig. 6:**
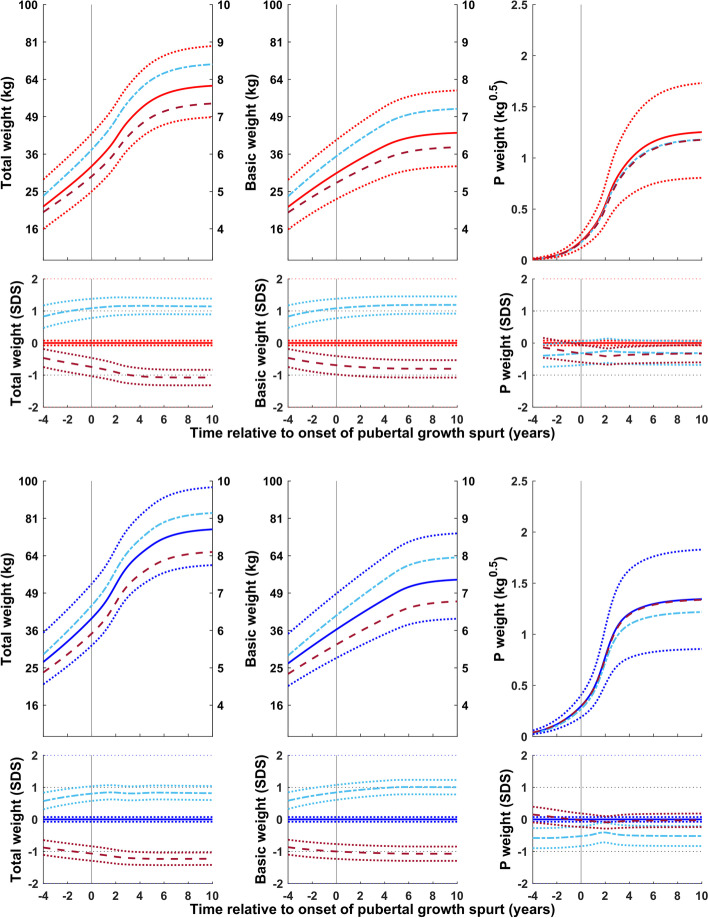
Total, basic, and puberty-specific weight gain (in kg, top; in SDS with 95%CI, bottom) relative to the onset of puberty for girls (red at the top) and boys (blue at the bottom) according to BMI in childhood (high BMI, > + 1.5SDS (- — -) and low BMI, <− 1.5SDS (- - -)). Mean total weight (left panels), basic growth (middle panels), and specific pubertal growth (right panels) in girls and boys from the GrowUp_1974_Gothenburg cohort. Curves are aligned for age at onset of pubertal growth spurt. Data are visualized alongside the new reference for total mean weight (thick red or blue solid lines) and ± 2SDS

Figure 6 shows weight (total (QEPS), basic (QES) and pubertal (P-function)) for girls and boys according to the highest BMI during childhood (3.5-7 yrs. in girls and 3.5-8 yrs. in boys)). The basic component of weight gain was higher than average in the high BMI group and lower than average in the low BMI group. Puberty-specific weight gain differed between the sexes: weight gain specific to the pubertal function was slightly lower than average in both the high and low BMI groups for girls, but in only the high BMI group for boys. Specific pubertal weight gain for boys in the low BMI group was similar to the average for the whole population.

### Using the new pubertal growth charts prospectively for an individual child

**Fig. 7:**
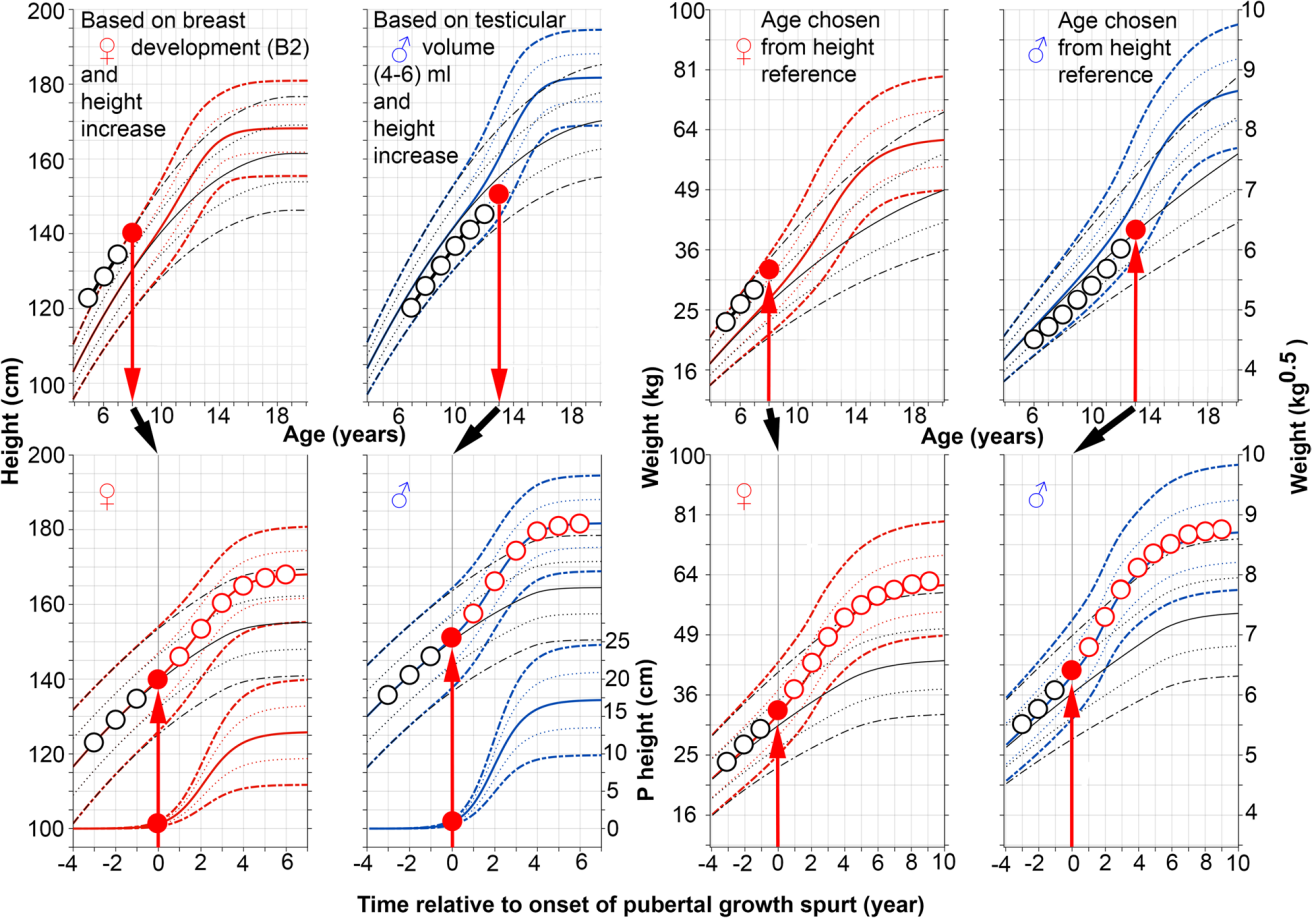
A guide for prospective use of the pubertal-age-adjusted reference for total weight (kg and kg^0.5^) and height (cm) for girls (**♀**) and boys (♂). Individual age adjustment is made only from the height measurement at the chronological age (C-age) at which puberty started (see upper left traditional C-age-reference for girls and boys) [14], by using the height increase from the individual prepubertal growth curve (as drawn in upper left panel) through the individual measuring points. This occurs when secondary sex characteristics develop; early breast development stage, B2 in girls (red, upper left panel), or testicular volume increase to 4–6 ml in boys (blue, upper left panel). Height and weight according to C-age and P-age, ie the age adjusted to reflect the start of puberty are shown for an 8-year-old girl and a 13-year-old boy [14]. Height (cm) and weight (in kg or kg^0.5^) according to C-age at the onset of puberty, and to P-age, after adjustment for age at onset of the pubertal growth spurt are depicted as red dots (all panels), based only on age in the height references for each sex respectively. Corresponding heights and weights are then moved to puberty-adjusted age = zero, in the P-age-references shown in the lower panels (left panels for height and right for weight). Thereafter, all measured heights and weights are depicted at ages/times recalculated in relation to the specific onset of puberty in that individual, labelled on the x-axis as ‘Time from onset of growth spurt (years)’. Thus, changes in weight and height in the years preceding the pubertal growth spurt can be evaluated using the novel reference. Note, the weight increase precedes the height increase in relation to puberty

A manual procedure can be undertaken in order to monitor pubertal growth prospectively for an individual child using the new pubertal-age-growth charts developed from the pubertal-age-adjusted reference. A growth chart with both total and prepubertal height [13], as well as the present weight references, should be used for easy identification of the ‘height-take off’ vs a prepubertal height reference at the start of puberty [13]. Figure 7 describes how to use the pubertal-age-adjusted reference prospectively to assess total weight.

## Discussion

### New prepubertal and pubertal weight references

We present the first pubertal weight references to be developed that can be adjusted to reflect the biological maturation of the individual child. The new references include median values and SDS that allow alignment of individual weight data based on the time/age at onset of the pubertal growth spurt. These references were created using the growth functions of the height QEPS-model which have previously been successfully applied to create a height reference that can be aligned for the onset of pubertal growth in the individual [14]. By enabling the clinician to simultaneously adjust both weight and height based on the maturational stage of the individual, these two references promise to improve the precision of the evaluation of growth during adolescence [14].

As well as enabling weight to be assessed relative to maturational stage, the weight references generated allow us to look separately at the prepubertal and pubertal components of weight gain. By separating weight gain related specifically to puberty (P-function) from other weight gain (QE-function), it is possible to look at ongoing, basic weight gain during adolescence that is unrelated to puberty. Thus, these references will form a novel tool with which to investigate both puberty-independent and puberty-dependent changes in weight.

### Weight changes precede height changes

By virtue of the alignment of weight data with the onset of the pubertal growth spurt, these references will allow us to look in more detail at the changes in weight that are known to precede the increase in height associated with the onset of puberty. Changes in weight before the acceleration of growth during puberty were already shown by Backman almost a century ago [17]. It is well established that pubertal growth begins with characteristic sex-specific changes in body fat mass, that occur earlier in girls than boys, and this suggests a link between energy storage in adipose tissue and pubertal maturation [18]. The new references will be new tool separating weight in relation to pubertal onset and by that increase our knowledge about changes in weight before the increase in height associated with puberty (Supplemental Fig. S3).

### Usefulness of a reference separating the growth functions during puberty

The benefit of using a growth model is not only to describe growth but also to be able to analyse how different growth functions can be related to the mechanisms underlying the regulation of growth [19, 20]. Here, thanks to the mathematical growth functions from the QEPS-weight and the QEPS-height models, we present a tool for investigating weight gain in new ways and with higher precision than previously possible. In this analysis, we explored differences in weight gain between groups of children with different characteristics prior to entering puberty. A novel finding from this analysis was that basic weight gain was greater in boys and girls who were late maturers or tall at the start of puberty, when compared to either the reference, or those of average pubertal timing or stature. The contrary, with lower than the average basic weight gain, was found for those who underwent early pubertal maturation or had short stature. P-function related weight gain was greater than the average in short individuals and lower than the average in tall individuals. Previously, we investigated children with different BMI, and found a positive relationship between BMI and greater than the average gains in basic height and lower than the average gains in puberty-specific height [21]. Results from the present study extend these findings, showing that a high BMI is also associated with greater than the average gains in basic weight and lower than the average gains in puberty-specific weight, and that low BMI is associated with lower than the average increases in basic weight. In addition, a sex-specific difference was found for specific pubertal growth, which was lower than the average in girls, and average in boys.

### A QEPS-weight model behind puberty-aligned weight references

Developing references for weight that are aligned for individual onset of puberty, require a growth model for individual weight. As only a model for weight in infancy and early childhood was available [22–25], we had to develop a model for the entire growth period that was good enough for this purpose. We also had to deal with the problem that weight measurements are known to be subject to greater daily variations than are expected for measurements of height. We therefore took the approach of transforming height values into corresponding values for weight in order to develop the weight model. Thus, the QEPS-weight model was supported by a transformation of fitted QEPS-height functions [11], into corresponding QEPS-weight functions. For better fitting results, introduction of only one individual factor was necessary; a specific individual weight parameter (*WHF*) in the weight model that was not predicted by height. However, the QEPS-weight model could also have been constructed and fitted directly on observed weight measurements using the six individual weight parameters, while optimizing the basic four shape-invariant QEPS-weight functions. In our experience, it would have resulted in considerable overfitting of the weight measurements, because of higher standard error of the fit for weight than for height. For height, the residual error was mostly owing to measurement error and time of day, whereas for weight, the residual variation was caused by many factors and could not simply be explained. Residual variation around individual predicted weight for normal body constitution might, for instance, also be related to individual variation in the relationship of fat /muscle distributions as estimated by body compartment analyses (DEXA) or subcutaneous fat measures, waist circumference or other comparable measurements. There are models for human growth and body dynamics [26]. Here, we have tried to find a good enough QEPS-weight model using the individual height information, however, other approaches or fine-tuning of the method remains to be developed.

### Biological correlates of individual WHF

The introduction of an individual WHF originated from the improved fitting of the model, described above and in Supplement. This gives rise to the question as to whether there is any biological correlate / connection to this factor. Adult height of an individual can fairly accurate be predicted by growth during infancy (ie attained height at 2 years of age), and delayed timing of transition from infancy to childhood growth corresponds to reduced adult height. Thus, it seems that there is a critical window in early life for changes in growth [19]. Moreover, clinically, there also appears to be a critical window during infancy for changing body size (correlate to WHF); it is known to be extremely difficult to change body size later on in life, if a child was thin (low WHF) or obese (high WHF) during infancy [27]. There is increasing evidence of a role for epigenetic programming during prenatal and early postnatal periods in determining child growth and development and later development of diseases. For instance, prenatal epigenetics have been shown to change IGF-I expression in several ways [28–30], and the Dutch Famine showed that famine during early and mid-gestation affects metabolism later in life, including increased risk for obesity [31]. There is also increasing evidence that variations in early postnatal nutrition have epigenetic effects on developmental programming and may result in the development of cardiovascular diseases, overweight, obesity, diabetes, and other chronic conditions [32–35].

### Prospective monitoring of growth in individual children using the new references and future perspectives

These new weight references will allow the creation of updated computerized references for use in healthcare settings. Until computerized versions of the puberty-aligned references for height and weight are available, a manual procedure can be undertaken to monitor pubertal growth prospectively for an individual child using the new references (see Fig. 7). A growth chart with both total and prepubertal height [13], as well as the present weight references, should be used for easy identification of the ‘height-take off’ vs the prepubertal height reference at the start of puberty [13]. The use of these new references will help to determine whether we should be concerned about the growth of late-maturing children who appear to be underweight, or early maturing children who appear to be overweight, based on assessments relative to chronological age. The availability of this type of reference is an important step towards more meaningful and informative monitoring of weight development of an individual during the adolescent years. The next step will be to develop references for other growth-related parameters, such as BMI.

## Conclusion

Here we present new types of weight references both for prepubertal weight in relation to chronological age, and for weight during pubertal years aligned for the onset of the pubertal growth spurt in the individual. When used together with the prepubertal and pubertal height references [13, 14], the new weight references will allow for improved growth monitoring during adolescence for detecting abnormal growth. The new references will also serve as valuable research tools that will help to provide new insights into human growth.

## Supplementary Information

**Additional file 1: Supplemental Material**.

## Data Availability

The generated and analyzed data during the current study are not publicly available but are available from the corresponding author on reasonable request.
