# Supplementary material for: Novel type of references for weight aligned for onset of puberty – using the QEPS growth model
Source: BMC Pediatr. 2021 Nov 13;21:507. doi: 10.1186/s12887-021-02954-z (PMC8590226; doi:10.1186/s12887-021-02954-z)
Supplement: Supplementary file 1 — Additional file 1: Supplemental Material. [file 12887_2021_2954_MOESM1_ESM.pdf]

Supplemental Material

To Albertsson-Wikland K et al: Novel type of references for weight aligned for onset of puberty - using the QEPS growth model

QEPS weight (wQEPS) growth model page 2

Figure S1: QEPS weight for normal, heavy and lean body constitution page 2

Fitting the wQEPS model with QEPS height functions page 2

Figure S2: QEPS height and predicted normal weight (*WHF*=0) page 3

Figure S3: Detailed QEPS pubertal growth estimates for height and weight page 4

wQEPS method for reference development page 5

Statistical evaluations page 5

QEPS weight growth model, with detailed mathematical equations

Summarizing the published QEPS height functions not adjusted with β page 6

Adjusting the hQEPS-functions with β and *WHF* page 6

Figure S2 beta: Figure S2 with nonlinear regression coefficients β added page 7

Fitting the QEPS weight model page 8

Abbreviations page 8

Figure S4: Total QEPS weight vs Total LMS weight page 10

REFERENCES page 10

QEPS weight (wQEPS) growth model

The QEPS height model (1) was transformed to a corresponding QEPS weight model. The QEPS-model for height and the corresponding QEPS-functions will be called hQEPS-model and hQEPS-functions to avoid confusion with respectively wQEPS-model and wQEPS-functions. The wQEPS-model for individual growth in weight was defined by a combination of four basic shape-invariant growth functions: a prepubertal quadratic *wQ*-function, a prepubertal negative exponential *wE*-function, a nonlinear pubertal *wP*-function, and a stop *wS*-function, describing total weight in $kg^{0.5}$ as a function of age:

$$wT(age|WHF) = wQ(age|WHF) + wE(age) + wP(age|WHF) - wS(age|WHF),$$

modified by an individual weight-height-factor (*WHF*) as shown in **Figure S1.** *WHF*=0 stands for a normal body constitution, *WHF*>0 for heavier and *WHF*<0 for leaner body constitution. The *WHF* has no influence on the *wE*-function. The *WHF* is a constant of proportionality with units ($kg^{0.5}/kg^{0.5}$). Multiplied with the predicted individual *QPS* weight function the *WHF* is quantifying the proportion of predicted *QPS* that is added to (*WHF* positive) or subtracted from (*WHF* negative) the predicted total weight function for normal body constitution to obtain the total weight function adjusted for constitution, so QEPS weight = (QEPS predicted weight) + (QPS predicted weight) * *WHF*. Written more formally the total individual weight function describing weight in $kg^{0.5}$ is: $wT(age|WHF) = wT(age|WHF=0) + wQPS(age|WHF=0) * WHF$. For a normal body constitution *WHF*=0 and the corresponding predicted total weight function $wT(age|WHF=0)$ is equal to the individual weight function as predicted by height. In the fitting procedure the median *WHF* of the model group was restricted to be zero to reflect the normal body constitution.

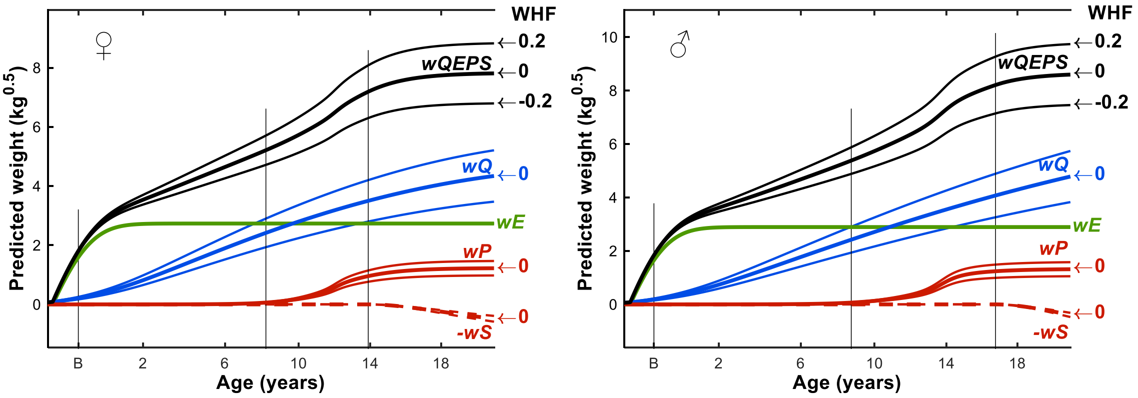

Figure S1: Total weight (*wQEPS*) in $kg^{0.5}$ for normal, heavy and lean body constitution with four growth functions *wQ*, *wE*, *wP* and *wS*.

wQ is a quadratic growth function, *wE* a negative exponential growth function, *wP* a pubertal growth function and *wS* a stop function modelling end of growth for function *wQ*. The weight-height-factor (*WHF*) can modify the growth functions *wQ*, *wP* and *wS*, but not *wE*. The *wQEPS*-function is shown for the typical mean “individual” weight function, as predicted by the typical mean height function of the QEPS height model (1), with normal (*WHF*=0, thick solid lines), heavy (*WHF*=0.2) and lean (*WHF*=−0.2) body constitution. The theoretical *WHF* values 0.2 (with 20% higher *wQ*, *wP*, *wS* function) and −0.2 (with 20% lower *wQ*, *wP*, *wS* function) are chosen within the observed range of fitted *WHF* values. Birth (B), start of *wP* and *wS*-function are marked with a vertical line. The age scale is transformed hyperbolically to stretch the early growth period.

Fitting the wQEPS model with QEPS height functions

The wQEPS model was fitted with customised hierarchical nonlinear regression on two levels:

-On group level individual QEPS weight was predicted from corresponding individual QEPS height functions as *hT(age, β)*, with 14 nonlinear regression coefficients, β_1 - β_{14}, common for all individuals within each gender of the model group.

On individual level the *hT(age, β)*-function was adjusted for individual body constitution by adding *hQPS(age, β)* * b_i, with b_i = *WHF*, the weight-height-factor for individual body constitution. The *β* are equal to the 14 regression coefficients modelled on group level.

In summary the wQEPS-model was specified with QEPS height functions as predictors:

$$wT(age|WHF) \qquad = hT(age, \beta) + hQPS(age, \beta) * WHF$$

In the fitting procedure the median *WHF* of the model group was restricted to be zero, so *wT(age|WHF*=0*)* = *hT(age, β)*, an individual's predicted weight for normal body constitution (see Figure S2). The *hP*-function was transformed in two *wP* subfunctions, *wP*=*wPA*+*wPB* (see Figure S3**).** A more detailed description on how the wQEPS-model was computed with nonlinear regression coefficients can be found on page 6 after "QEPS weight growth model, with detailed mathematical equations".

Figure S2

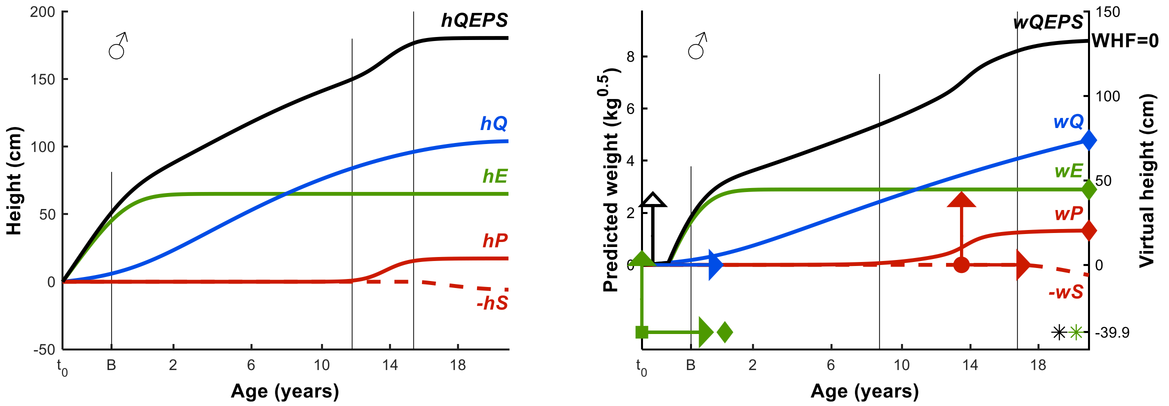

Figure S2: QEPS height and predicted normal weight (*WHF*=0)

Transforming individual QEPS height functions, *hQEPS*, for boys (left panel) into individual predicted weight functions for normal body constitution (*wQEPS(age|WHF*=0*)*, right panel) using *wQEPS(age|WHF*=0*)*=*hQEPS(age, β)*, with 14 nonlinear regression coefficients, β_1 - β_{14}, common for all individuals within each gender of the model group. The arrows in the right panel modify the time and height scale of the QEPS height functions. The horizontal arrows from left to right change the time-scale of the *hE*, *hQ* and *hP*-functions with 3+1 coefficients, since the *hP*-function is transformed in two *wP* subfunctions, *wP*=*wPA*+*wPB* (see Figure S3). Likewise, the height-scale of the *hE* and *hP*-functions indicated with vertical solid arrows is changed with 2+1 coefficients. The solid green square indicates the upwards shift of the origin of the *hE*-function. The solid red circle indicates the mid puberty shift at *hAgeP50* for the *wPA*-function. The previous 9 coefficients define the typical shape of the QEPS weight functions, common to all individual *wQ*, *wE*, *wP* and *wS* functions. The four solid diamonds adjust the deviation from typical mean parameter value 1 for individual estimated QEPS height scale parameters $Q_{heightscale}$, $E_{heightscale}$, $P_{heightscale}$, and $E_{timescale}$. The previous 13 coefficients define a kind of intermediate *QEPS* virtual height functions (in virtual cm, right panel right-hand scale with origin negative for *virtualheightE* and *virtualheightQEPS*-function). The 14th coefficient gives the conversion to predicted normal weight (kg$^{0.5}$, left-hand scale) by changing the scale of all *virtualheightQEPS*-functions as indicated by the black open vertical arrow. Birth (B), start of *wP* and *wS*-function are marked with a vertical line. Notice the later start of *wE*-function growth, and the longer duration and earlier onset of puberty for weight compared to height.

Figure S3

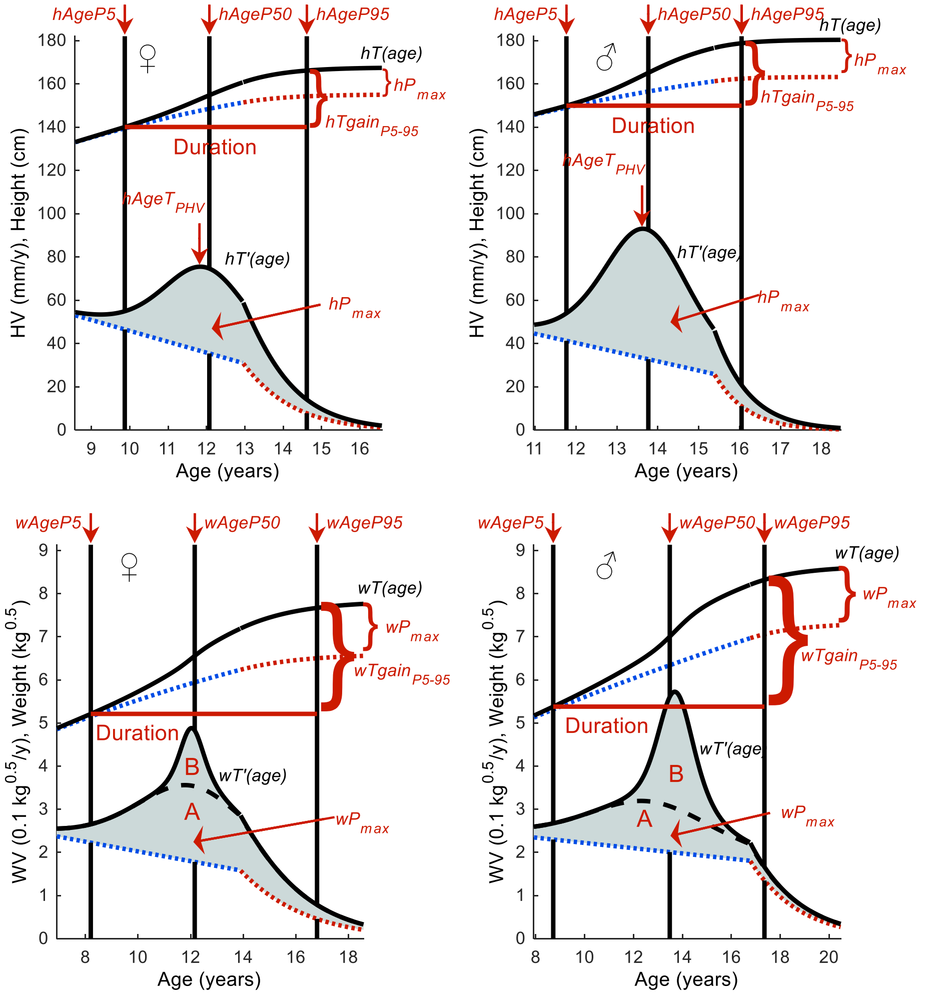

Figure S3: Detailed QEPS pubertal growth estimates for height (upper panels) and weight (lower panels) for girls and boys during puberty

Typical mean growth functions during puberty for height and height velocity (upper panels) and weight and weight velocity (lower panels) for girls (left panels) and boys (right panels). Onset, *hAgeP5* for height and *wAgeP5* for weight, as age at which 5% of the *P*-function growth is reached, mid puberty, *hAgeP50* for height and *wAgeP50* for weight, as age at which 50% of the *P*-function growth is reached and Age at Peak Height Velocity $hAgeT_{PHV}$, of the total height function *hT* from the QEPS height model is reached and end of pubertal growth, *hAgeP95* for height and *wAgeP95* for weight, as age at which 95% of the *P*-function growth is reached are marked with vertical lines. The duration of puberty for height and weight is indicated with horizontal lines. The pubertal height or weight gain is shown as the growth from *AgeP5* to *AgeP95* from the total growth curve ($Tgain_{P5\text{-}95}$), and from the specific pubertal part (*Pmax*); both shown at the top of the figure in the height and weight graphs and as the marked area in the height velocity and weight velocity graphs. The marked areas in the weight velocity graphs show that *wP* is a sum of two subfunctions, *wPA* and *wPB*. The *wPB* subfunctions for both girls and boys were restricted in the fitting procedure to have *wAgePB50* equal to *hAgeP50*. For girls *wPBmax* is 13% of *wPmax* and for boys 41% of *wPmax*.

wQEPS method for reference development

References for age were constructed for both total weight and prepubertal weight and were computed for the QEPS weight reference population in two steps comparable to the 'QEPS method' used for the references (2, in corresponding supplement) with QEPS height functions here replaced by corresponding QEPS weight functions. Special pubertal growth-aligned references *(wQEPSpub=wTpub*, *wQESpub* and *wPpub)* were constructed by aligning individual function values for *wT(age)*, *wQES(age)* and *wP(age)* by the individual age at onset of the pubertal growth spurt, defined as *hAgeP5*, the age at which 5% of height specific *hP*-function growth was reached (see Figure S3, upper panels).

Statistical evaluations

Longitudinal growth data for each individual, including height and weight measurements were exported to Matlab®(version 9.3 R2017b, The MathWorks). The Matlab Curve Fitting Toolbox was used for estimating individual QEPS height parameters by nonlinear fitting. The parameters of the wQEPS model were also fitted with Matlab. The standard error of the residuals was used to assess goodness-of-fit. To simplify general mathematical formulae, age was assumed to be equal to age corrected for GA, here 37–43 weeks. Figure 7 was prepared with SAS software, version 9.3, all other figures with Matlab.

QEPS weight growth model, with detailed mathematical equations

The QEPS height model (1) was transformed to a corresponding QEPS weight model. The QEPS-model for height and the corresponding QEPS-functions will be called hQEPS-model and hQEPS-functions to avoid confusion with respectively wQEPS-model and wQEPS-functions. The wQEPS-model for individual growth in weight was defined by a combination of four basic shape-invariant growth functions: a prepubertal quadratic *wQ*-function, a prepubertal negative exponential *wE*-function, a nonlinear pubertal *wP*-function, and a stop *wS*-function, describing total weight in $kg^{0.5}$ as a function of age:
$wT(age|WHF) = wQ(age|WHF) + wE(age) + wP(age|WHF) - wS(age|WHF)$,
modified by an individual weight-height-factor (*WHF*). The wQEPS model was fitted with customised hierarchical nonlinear regression on two levels:
-On group level individual QEPS weight was predicted from corresponding individual QEPS height functions as $hT(age, \beta)$, with 14 nonlinear regression coefficients, β_1 - β_{14}, common for all individuals within each gender of the model group.
-On individual level the $hT(age, \beta)$-function was adjusted for individual body constitution by adding $hQPS(age, \beta) * b_i$, with $b_i = WHF$, the weight-height-factor for individual body constitution. The β are equal to the 14 regression coefficients modelled on group level.
In summary the wQEPS-model was specified with QEPS height functions as predictors:

$$wT(age|WHF) = hT(age, \beta) + hQPS(age, \beta) * WHF$$

In the fitting procedure the median *WHF* of the model group was restricted to be zero, so $wT(age|WHF{=}0) = hT(age, \beta)$, an individual's predicted weight for normal body constitution.

Summarizing the published QEPS height functions not adjusted with β

The hQEPS-functions not adjusted with β gave QEPS total height in cm as a function of age:

$$hT(age) = hQ(age) + hE(age) + hP(age) - hS(age).$$

The core of the QEPS height growth model was the typical mean growth function $mhT(age) = mhQ(age) + mhE(age) + mhP(age) - mhS(age)$, with four typical mean growth functions *mhQ*, *mhE*, *mhP* and *mhS*, having a shape common to all individual functions *hQ*, *hE*, *hP* and *hS*. To fit observed height measurements, six parameters were estimated with separate curve fitting for each individual: four height-scale parameters, $Q_{heightscale}$, $E_{heightscale}$, $P_{heightscale}$, $S_{heightscale}$ modifying the scale of the y-axis of respectively *mhQ*, *mhE*, *mhP* and *mhS*; and two time-scale parameters, $hE_{timescale}$ and $hP_{timescale}$ modifying the scale of the age-axis of respectively *mhE* and *mhP*. The height scale parameter $S_{heightscale}$ was a function of the individual mid puberty parameter *hAgeP50* (for known $Q_{heightscale}$ and $hP_{timescale}$) and was replaced by this parameter in the equations.
The QEPS typical mean height parameter values for [Girls; Boys] are:

hQ_{linear}=[9.949738642531; 9.405436598935], hQ_{quadr}=[−0.2514723125035; −0.2125041669303],
t_0=[−0.6627671903241; −0.6494334970962],
mhE_{max}=[62.54785757042; 65.04702921079], hE_k=[1.823677860659; 1.833525449549].
mhP_{max}=[12.5200654; 17.2388724], hP_{linear}=[1.25295488; 1.38636865], hP_{quadr}=[0.0236603572; 0.0217097456],
mhAgeP50=[12.0742839; 13.7679612],
$mhAgeS_0$=[12.95101524; 15.379483666], $mhAgeS_{max}$=[14.2767288; 16.198706],
and the estimated individual height parameters are $Q_{heightscale}$, $E_{heightscale}$, $hE_{timescale}$, $P_{heightscale}$, $hP_{timescale}$ and *hAgeP50*.

Adjusting the hQEPS-functions with β and *WHF*

Four basic shape-invariant wQEPS-functions could be expressed as adjusted hQEPS- functions as follows:

$$wQ(age|WHF) = hQ(age, \beta) + hQ(age, \beta) * WHF,$$
$$wE(age) = hE(age, \beta),$$
$$wP(age|WHF) = hP(age, \beta) + hP(age, \beta) * WHF,$$
$$wS(age|WHF) = hS(age, \beta) + hS(age, \beta) * WHF.$$

The nonlinear regression coefficients β are specified below, numbered β_1 - β_{14} and followed by an acronym indicating which function is adjusted. The estimated values for [Girls; Boys] are:

β_1=*βThsc*=0.5*[0.09524013;0.1326592];
β_2=*βQhdev*=[1.411883;1.665129]; β_3=*βQtsc*=[1.473111;2.179397];
β_4=*βEhdev*=[0.5727472;0.5374842]; β_5=*βEhsc*=[1.594266;1.219128];
β_6=*βEtdev*=[0.715742;0.7393889]; β_7=*βEtsc*=[0.8399047;0.8153451];
β_8=*βE0shift* =[42.15111;35.59802];
β_9=*βPhdev*=[0.5273259;0.5826083]; β_{10}=*βPhsc*=[2.053879;1.158501];
β_{11}=*βAgePA50shift*=[0.1376324;-1.027676]; β_{12}=*βPAtsc*= β_3 * [1.299556;1.142874];
β_{13}=*βPBhsc*=[0.1324334;0.4074699]; β_{14}=*βPBtsc*=[0.3846266;0.6856515];
with *T=Q+E+P –S*, *hsc=heightscale*, *hdev=deviation* from *hsc*=1, *E0shift=shift E zero point,*
P=PA+PB, *tsc=timescale*, *tdev=deviation* from *tsc*=1, *AgePA50shift*=Mid puberty shift of *PA*-function

Figure S2 beta

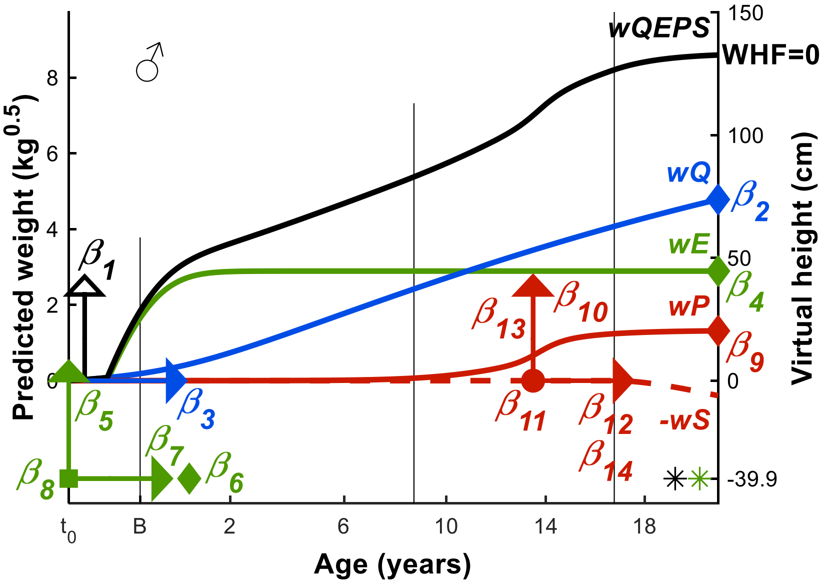

Figure S2 beta: Figure S2 with nonlinear regression coefficients β added.

The *hQ*-function was adjusted with nonlinear regression coefficients β_1, β_2, β_3:
$hQ(age, \beta) = \beta_1\, Q_{weightscale}\, mhQ(age, \beta_3)$
with $Q_{weightscale} = 1 + \beta_2\, (Q_{heightscale} - 1)$
and $mhQ(age, \beta_3) = wQ_{linear}\, (ageQ_{virtual} - t_0) + wQ_{quadr}(\beta_3)\, (ageQ_{virtual} - t_0)^2$

with $ageQ_{virtual} = (age \leq wAgeQ_{max})\; age + (age > wAgeQ_{max})\; wAgeQ_{max}$,
$wQ_{linear} = hQ_{linear} / \beta_3$ and $wQ_{quadr} = hQ_{quadr} / \beta_3^2$,
and $wAgeQ_{max} = t_0 - wQ_{linear}/(2wQ_{quadr})$.

The *hE*-function was adjusted with coefficients β_1, β_4, β_5, β_6, β_7, β_8:
$hE(age, \beta) = \beta_1 \left(E_{weightscale}\, mhE(age, hE_{timescale}, \beta_5, \beta_6, \beta_7) - \beta_8\right)$
with $E_{weightscale} = 1 + \beta_4\, (E_{heightscale} - 1)$and $mhE(age, hE_{timescale}, \beta_5, \beta_6, \beta_7) = \beta_5\, mhE_{max} \left(1 - e^{-wE_k k \frac{age - t_0}{wE_{timescale}}}\right)$
with $wE_k = hE_k / \beta_7$ and $wE_{timescale} = 1 + \beta_6\, (hE_{timescale} - 1)$.
Function $hE(age, \beta)$ was restricted to be nonnegative by setting all negative values to zero.

The *hP*-function was adjusted with coefficients β_1, β_9, β_{10}, β_{11}, β_{12}, β_{13}, β_{14}:
$hP(age, \beta) = \beta_1\, P_{weightscale}\, mhP(age, hAgeP50, hP_{timescale}, \beta_{10}, \beta_{11}, \beta_{12}, \beta_{13}, \beta_{14})$

with $P_{weightscale} = 1 + \beta_9 \, (P_{heightscale} - 1)$
and $mhP(age, hAgeP50, hP_{timescale}, \beta_{10}, \beta_{11}, \beta_{12}, \beta_{13}, \beta_{14}) = \beta_{10} \, mhP_{max} \, *$

$$\Bigg((1-\beta_{13}) \frac{1}{1+e^{wPA_{linear}\frac{wAgePA50-age}{hP_{timescale}} + hP_{quadr}\left(wPA_{linear}\frac{wAgePA50-age}{hP_{timescale}}\right)^2}} + \beta_{13} \frac{1}{1+e^{wPB_{linear}\frac{wAgePB50-age}{hP_{timescale}} + hP_{quadr}\left(wPB_{linear}\frac{wAgePB50-age}{hP_{timescale}}\right)^2}} \Bigg)$$

with $wAgePA50 = hAgeP50 + \beta_{11} \, hP_{timescale}$ and $wAgePB50 = hAgeP50$
and $wPA_{linear} = hP_{linear} / \beta_{12}$ and $wPB_{linear} = hP_{linear} / \beta_{14}$.

The *hS*-function was adjusted with coefficients $\beta_1, \beta_2, \beta_3, \beta_{11}, \beta_{12}$:

$$hS(age, \beta) = -\beta_1 \, Q_{weightscale} \, wQ_{quadr} \, d_{sq}^2 \left(1 - \left(\frac{ageQ_{virtual} - ageS_{virtual})}{d_{sq}} - 1\right)^2\right)$$

with $Q_{weightscale}$, wQ_{quadr}, $ageQ_{virtual}$, $wAgeQ_{max}$ as defined for $hQ(age, \beta)$,
$d_{sq} = wAgeQ_{max} - wAgeS_{max}$,
with $wAgeS_{max} = wAgePA50 + hP_{timescale} \, \beta_{12} \, (mhAgeS_{max} - mhAgeP50)$,
with $wAgePA50$ as defined for function $hP(age, \beta)$,
and $ageS_{virtual} = (age \leq wAgeS_0) \, age +$

$$(age > wAgeS_0) \left(wAgeS_0 + hP_{timescale} \, c \left(1 - e^{-\frac{age - wAgeS_0}{hP_{timescale} \, c}}\right)\right),$$

with $wAgeS_0 = wAgePA50 + hP_{timescale} \, \beta_{12} \, (mhAgeS_0 - mhAgeP50)$
and $c = \beta_{12} \, (mhAgeS_{max} - mhAgeS_0)$.

The resulting $hT(age, \beta)$-function, giving individual total QEPS weight predicted from corresponding QEPS height, was adjusted for individual body constitution by adding $hQPS(age, \beta) * b_i$, with $b_i = WHF$, the weight-height-factor for individual body constitution.

Fitting the QEPS weight model

The nonlinear regression coefficients β were estimated by minimizing the residual sum of squares:

$$SSE = \sum_{i=1}^{n} \| \mathbf{wT}_{\mathbf{i}Observed} - \mathbf{hT_i}(\mathbf{x_i} | \beta) - \mathbf{hQPS_i}(\mathbf{x_i} | \beta) \, b_i \|^2,$$

for vector columns $\mathbf{wT}_{\mathbf{i}Observed}$ with n_i observed weights, vector columns $\mathbf{hT_i}(\mathbf{x_i}|\beta)$ and $\mathbf{hQPS_i}(\mathbf{x_i}|\beta)$ with corresponding n_i function values at visit ages $\mathbf{x_i}$ and individual weight-height-factor *WHF* computed as,

$$b_i = \frac{(\mathbf{wT}_{\mathbf{i}Observed} - \mathbf{hT_i}(\mathbf{x_i}|\beta))' \, \mathbf{hQPS_i}(\mathbf{x_i}|\beta)}{\mathbf{hQPS_i}(\mathbf{x_i}|\beta)' \, \mathbf{hQPS_i}(\mathbf{x_i}|\beta)}.$$

In the fitting procedure the median b_i of the model group (n=1779 girls and n=1816 boys) was restricted to be zero by computing $b_{geomean} = (\Pi \, (1+b_i))^{1/n} - 1$ and adjusting $b_i = b_i - b_{geomean}$ for all i. Taken into account that (1+*WHF*) is lognormal distributed and the geometric or multiplicative mean of the log-normal distribution equals the median. The standard error of the residuals was for girls 0.12207 $kg^{0.5}$ (*SSE*=748.41, *df*=50225) and for boys 0.11917 $kg^{0.5}$ (*SSE*=706.81, *df*=49771).

Abbreviations

E	negative exponential growth function of age, *hE(age)* for height and *wE(age)* for weight
Eheightscale	individual height-scale ratio, modifying the height-scale of the *hE*-function growth
Emax	*hEmax* gain in adult height due to *hE*-function and *wEmax* gain due to w*E*-function growth
Eweightscale	individual weight-scale ratio, modifying the weight-scale of the *wE*-function growth
hAgeP5	age at which 5% of height *hP*-function growth is reached
hAgeP50	age at which 50% of height *hP*-function growth is reached
hAgeQmax	age when the maximum of the *hQ*-function growth is reached: for girls at 19.1 years and for boys at 21.5 years

hAgeT$_{PHV}$	age at Peak Height Velocity of the total height function *hT*
hEtimescale	individual time-scale ratio; modifying the time-scale of the *hE*-function growth and therefore inversely related to the tempo of *hE*. The origin is at t_0, the age when length is theoretically zero, $hE(t_0)$=0, $hQ(t_0)$=0
GA	gestational age
P	function describing the pubertal growth, as *hP(age)* for height and *wP(age)* for weight
Pheightscale	individual height-scale ratio, modifying the height-scale of the *P*-function
Pmax	*hPmax* pubertal gain in adult height due to the *hP*-function growth and *wPmax* pubertal gain due to w*P*-function growth
hPtimescale	individual time-scale ratio, modifying the time-scale of the *hP*-function and is therefore inversely related to the tempo of *hP*. The origin is at *hAgeP50*, the age at which 50% of the individual *hP*-function growth is reached
Q	quadratic growth function of age, specified as *hQ(age)* for height and *wQ(age)* for weight
QE	sum of *Q* and *E*-function giving non pubertal growth *QE(age)*, excluding puberty (*P* and *S* functions) equal to total prepubertal height (*hQE*) or weight (*wQE*) as a function of age; the growth during prepubertal years specified as *hQE(age)* for height and *wQE(age)* for weight
QES	*QE* growth stopped at the end of puberty by *S*-function, with *QES(age) = QE(age) - S(age)*, also called basic growth function, specified as *hQES(age)* for height and *wQES(age)* for weight
QESmax	gain in adult height or weight due to *QES*-function growth, so *QESmax* = *QEmax* – *SmaxS*, specified as *hQESmax* for height and *wQESmax* for weight
Qheightscale	individual height-scale ratio, modifying the height-scale of the *hQ*-function
Qmax	*hQmax* gain in adult height due to *hQ*-function and *wQmax* gain in adult weight due to w*Q*-function growth
Qweightscale	individual height-scale ratio, modifying the weight-scale of the *wQ*-function
S	stop function *S(age)*, stopping the *Q*-function at the end of growth, specified as *hS(age)* for height and *wS(age)* for weight
Smax	reduction in adult height or weight due to stopping the *Q*-function growth at the end of growth, specified as *hSmax* for height and *wSmax* for weight
T	total height or weight function: *T(age)* = *QEPS(age)* = *Q(age)* + *E(age)* + *P(age)* − *S(age)*, specified as *hT(age)* for height and *wT(age)* for weight
t_0	age when length is theoretically zero, $T(t_0)$=0, $E(t_0)$=0, $Q(t_0)$=0, at about −0.66 years for girls and −0.65 years for boys
Tmax	modelled total adult height, *hTmax* = *hEmax* + *hQmax* + *hPmax* – *hSmax* and modelled total adult weight, *wTmax* = *wEmax* + *wQmax* + *wPmax* – *wSmax*
wAgeP5	age at which 5% of height *wP*-function growth is reached
wAgeP50	age at which 50% of height *wP*-function growth is reached
wPtimescale	individual time-scale ratio, modifying the time-scale of the *wP*-function and is therefore inversely related to the tempo of *wP*. The origin is at *wAgeP50*, the age at which 50% of the individual *wP*-function growth is reached

Figure S4

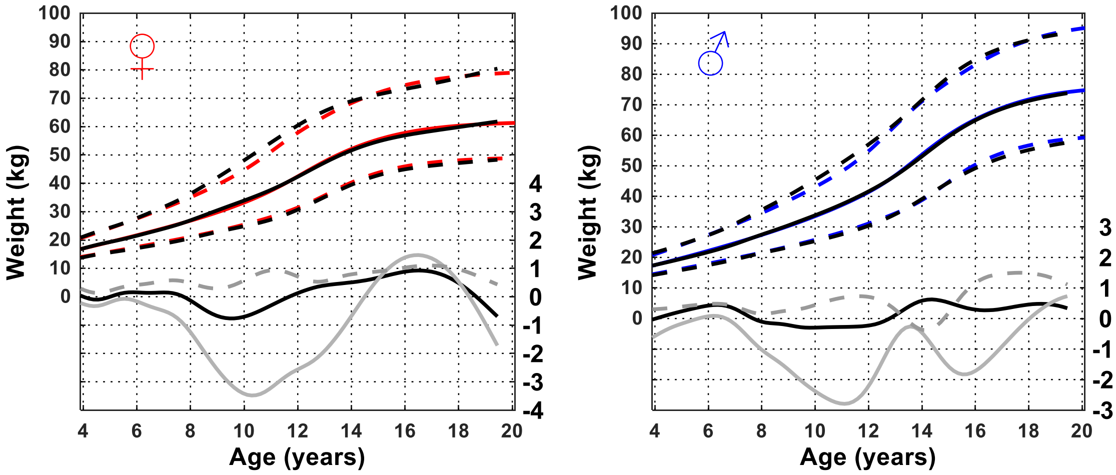

Figure S4: Total QEPS weight vs Total LMS weight
Comparison of the total 1990 QEPS weight reference (red/blue) with the published 1990 LMS weight reference (black).
Total weight references for children aged 4-18 years are shown for girls on the left and for boys on the right. The difference between the total weights obtained by the two methods, QEPS-derived or LMS-derived, are shown in the bottom of each figure. Differences in mean (thick solid black line), in +2SDS (solid gray line) and in −2SDS (dotted gray line) are shown.
Visual inspection shows that, although the mean curves based on the QEPS and LMS methods are quite similar, lines representing ±2SDS of the QEPS-derived reference are closer to the mean curves than lines of the LMS-derived reference. The variation of the QEPS-derived reference is smaller since it is based on fitted weight functions, excluding all sorts of weight error, whereas the LMS-derived reference is fitted on observed weight measurements, including such error.

REFERENCES

1. Nierop AF, Niklasson A, Holmgren A, Gelander L, Rosberg S, Albertsson-Wikland K. Modelling individual longitudinal human growth from fetal to adult life - QEPS I. J Theor Biol. 2016; 406:143-65
2. Albertsson-Wikland K, Niklasson A, Holmgren A, Gelander L, Nierop AFM. A new Swedish reference for total and prepubertal height. Acta Paediatr. 2020; 109:754-63
